# Value-Based Pricing: L’Enfant Terrible?

**DOI:** 10.1007/s40273-017-0567-4

**Published:** 2017-12-21

**Authors:** Sarah Garner, Andrew Rintoul, Suzanne R. Hill

**Affiliations:** 1 Innovation, Access and Use, Department of Essential Medicines and Health Products, World Health Organization, Geneva, Switzerland; 2 Department of Essential Medicines and Health Products, World Health Organization, Geneva, Switzerland

Concern over pricing of pharmaceuticals and other health technologies in both high- and low-income countries is not new. It has been high on the World Health Organization (WHO) agenda for a number of years [1]. Affordability of products, both to individual patients and to health systems, is one of the main barriers to accessing many effective medicines. In high income countries this debate has been focused primarily on medicines for cancer and orphan diseases, but in 2014 the pricing of sofosbuvir expanded the issue much more broadly: here was a ‘cost-effective’ treatment for hepatitis C that was unaffordable to countries of any income. The price being asked on the basis of cost-effectiveness evaluations might be considered to be ‘value based’, but as described in Iyengar et al. [2], was completely unaffordable for countries to use to treat all eligible patients. So what has gone wrong with so-called value-based pricing (VBP)?

VBP is a well-established pricing strategy for commodities. The basic idea behind this approach is that the price of goods should reflect the value to the buyer rather than the actual costs of production plus a margin. But in the context of pharmaceuticals there is no widely accepted definition of VBP. It is generally defined as the use of any policy or strategy designed to link the price and/or approval of a pharmaceutical or health product to the perceived value of the product [3]. On the face of it the theory is very simple; health systems should pay similar amounts for products with the same therapeutic effect or ‘value’. However, in practice this has proved more complicated, particularly when establishing what metrics should be included in ‘value’ assessments.

As noted by Neyt [4], assessments of the clinical and economic value of pharmaceuticals are a key component in many countries for decisions as to whether specific pharmaceuticals should be provided by health care systems. Indeed, health technology assessment and cost-effectiveness evaluation have been used by high-income countries for over 20 years, notwithstanding the well-documented limitations of economic evaluations [5–7]. As Neyt also points out, there have been many debates about using ‘cost-effectiveness thresholds’. But we have to emphasise that WHO has *not* recommended three times gross domestic product (GDP)/capita as ‘relatively cost effective’ [8], which highlights that even in high-income countries controversy and confusion reigns over what the cost-effectiveness threshold represents. Is it the ‘shadow price’ or does it represent ‘willingness to pay’? As Neyt correctly says, the cost-effectiveness ratio should only be the first consideration of many in making decisions about what to reimburse. But what we have learnt is that using cost-effectiveness ratios as the sole basis of either decision making or price setting is fraught with difficulties. For example, should ‘innovation’ attract a premium regardless of the actual therapeutic benefits? Industry has argued for this approach to ensure continued innovation [9], but determining how much innovation is worth is as difficult as determining ‘value’; particularly when the research and development costs are not transparent. 

Globally, the risks of using value-based assessments as the sole basis for pricing are that it does not take into account need, prevalence and affordability. As the Lancet Commission on Essential Medicines [10] pointed out, affordability is “distinct from the value of a product or service. Thus, an essential medicine might offer a large health benefit or high value (determined, for example, through cost-effectiveness analysis), but still might not be affordable (because of limited resources, high prices, or both)…”. So given the technical challenges of using health technology assessment, focussing solely on VBP also has the potential to undermine existing and effective systems of competitive tendering and price-volume agreements. These are vital components of many countries’ purchasing strategies, regardless of income.

WHO supports its 194 Member States as they coordinate the efforts of multiple sectors of the government and partners—including bi- and multilaterals, funds and foundations, civil society organizations and private sector entities—to attain their health objectives and support their national health policies and strategies. The profile of these 194 Member States is extremely varied: in relation to pharmaceuticals it ranges from high-income countries with established health technology assessment and guideline systems to low-income countries without an existing supply chain or technical skills. In some countries the predominant pharmaceutical purchasing system is competitive tendering and price-volume agreements, based on quality generic products and an essential medicines list. There is global interest in using health technology assessment to inform prices but it has to work in conjunction with these vital existing mechanisms.

In 2014 the WHO Member States formally requested that WHO’s Director General, through World Health Assembly Resolution WHA67.22 (“Access to Essential Medicines”, 2014), support countries to “ensure access to safe, effective and quality-assured essential medicines, including high price essential medicines.” At the same Assembly, another resolution was passed on “Health intervention and technology assessment in support of universal health coverage.” In order to support both resolutions, the Fair Pricing Initiative was launched leading to the “Fair Pricing Forum”. The main aim of the Forum was to enable stakeholders to discuss options for a fairer pricing system that is sustainable for both health systems and the pharmaceutical industries. The Forum sought to address three questions: What can governments do to ensure fairer medicines prices and greater access? What can industry do? How can WHO support the process [11]?

The outcome of the Forum is that there is much to do to agree on how a fairer pricing model can be achieved that ensures access to medicines without bankrupting progress towards universal health coverage. Comparative effectiveness assessment and budget impact evaluation by decision makers will remain critical tools going forward, and there we agree with Neyt and many others about using evidence to fully inform decisions. But equally important is the need to change the rhetoric about what constitutes a fair and sustainable price for all—and that must start with transparency of R&D costs and expected return on investment rather than just discussion of value. In the end, there is no value in a medicine that is too expensive and sits on the shelf.
